# Patent foramen ovale revealed by COVID-19 pneumonia

**DOI:** 10.1186/s12890-021-01494-7

**Published:** 2021-04-19

**Authors:** Charlotte Vanhomwegen, Olivier Taton, Nicolas Selvais, Olivier Vanhove, Dimitri Leduc

**Affiliations:** 1 Present Address: CHU Erasme Hospital, Université Libre de Bruxelles, Route de Lennick 808, 1070 Brussels, Belgium; 2 Department of Pneumology, CHU Erasme Hospital, Université Libre de Bruxelles, Bruxelles, Belgium; 3 Department of Cardiology, CHU Erasme Hospital, Université Libre de Bruxelles, Bruxelles, Belgium

**Keywords:** Hypoxemia, SARS-CoV-2, Patent foramen ovale, Pulmonary vasoconstriction, Ventilation inhomogeneity

## Abstract

**Background:**

Platypnea-orthodeoxia syndrome (POS) is a rare condition characterized by dyspnoea (platypnea) and arterial desaturation in the upright position resolved in the supine position (orthodeoxia). Intracardiac shunt, pulmonary ventilation–perfusion mismatch and others intrapulmonary abnormalities are involved.

**Case presentation:**

We report a case of POS associated with two pathophysiological issues: one, cardiac POS caused by a patent foramen ovale (PFO) and second, pulmonary POS due to severe acute respiratory syndrome coronavirus 2 (SARS-CoV-2) interstitial pneumonia. POS has resolved after recovery of coronavirus disease 2019 (COVID-19) pneumonia.

**Conclusions:**

Right-to-left interatrial shunt and intrapulmonary shunt caused by SARS-CoV-2 pneumonia contributed to refractory hypoxemia and POS. Therefore, in case of COVID-19 patient with unexplained POS, the existence of PFO must be investigated.

## Background

COVID-19 causes an atypical acute respiratory distress syndrome (ARDS) and becomes pandemic [1, 2]. Hypoxemia is the mean feature of SARS-CoV-2 pneumonia and results from several pathologic ways that are not completely understood [3].

Positional hypoxemia related to POS is caused by right-to-left shunting (RTLS) bypassing pulmonary oxygenation due to intracardiac or intrapulmonary abnormalities. It is defined by a drop in oxygen saturation greater than 5% from supine to upright position [4]. PFO is common in adult population and is harmless for most of the time except under pathologic conditions [5]. We present a COVID-19 patient who presented refractory positional hypoxemia associated with unknown PFO.

## Case presentation

A 55-year-old man presented in emergency department with fever and dyspnoea for 1 week. His medical history was notable for kidney transplant 2 years ago and he was treated with tacrolimus (blood level on admission 13 µg/L, normal range 5–7 µg/L), mycophenolate mofetil (750 mg twice a day) and methylprednisolone (4 mg once a day).

**Fig. 1:**
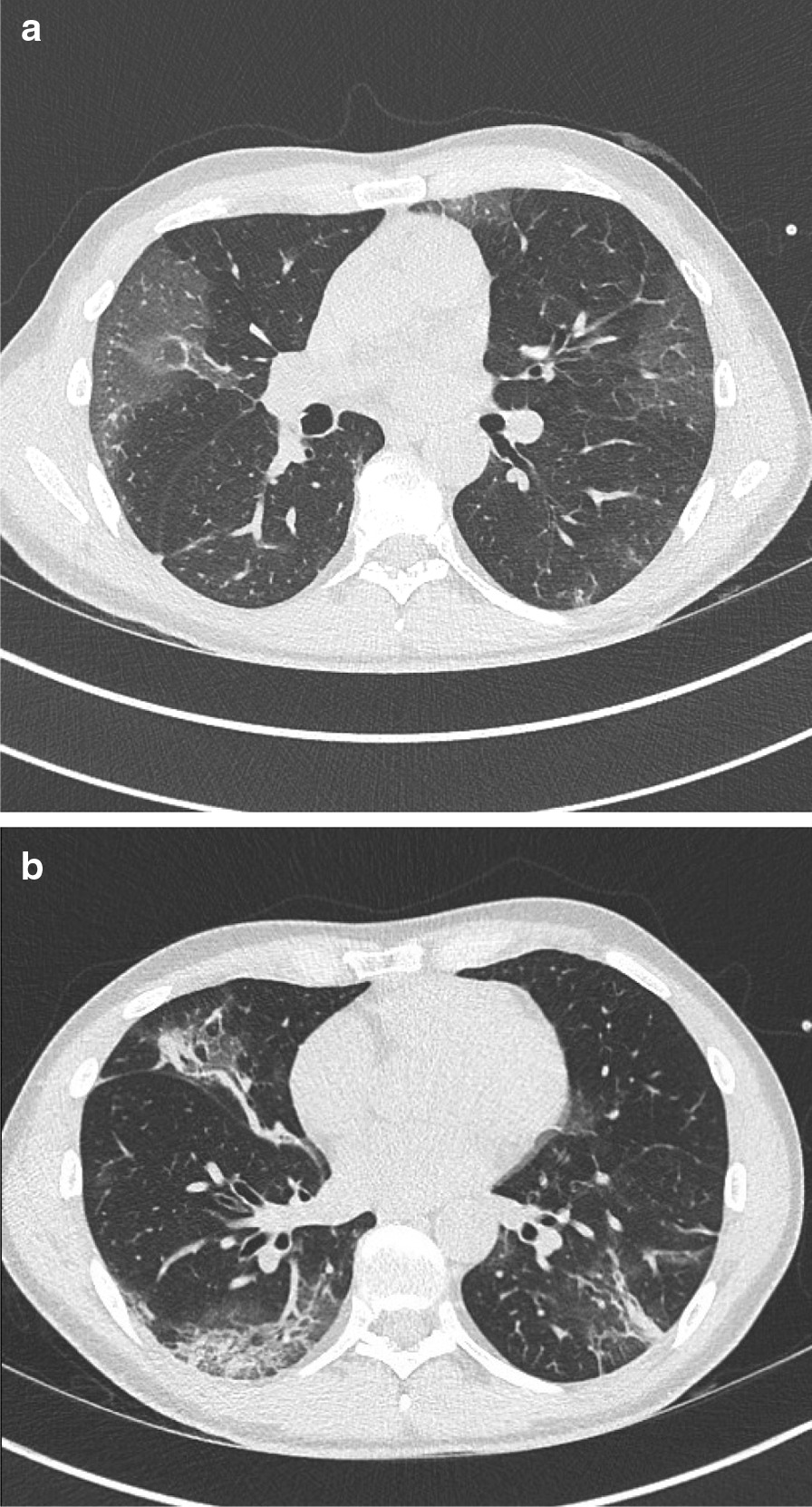
CT scan on admission. **a** Bilateral ground glass opacities predominantly with a peripheral lung distribution. **b** Crazy paving pattern in the right middle lobe and in the inferior lobes bilaterally

The arterial blood gas analysis at room air showed PaO2 60 mmHg, PaCO2 22 mmHg, pH 7.40 and P/F ratio 286. Creatinine at admission was 1.63 mg/dL (normal range 0.70–1.20). ECG showed no abnormalities. The patient had a positive swab for SARS-CoV-2 by reverse transcriptase polymerase chain reaction (RT-PCR). Chest Computed tomography (CT) scan showed bilateral peripheral ground glass opacities with crazy paving patterns (Fig. 1). He was hospitalized in the Middle Care Unit and was treated with oxygen therapy and Boussignac continuous positive airway pressure (BCPAP, PEEP 3 cmH2O, FiO2 50%) and dexamethasone (6 mg once a day) for ten days.

**Fig. 2:**
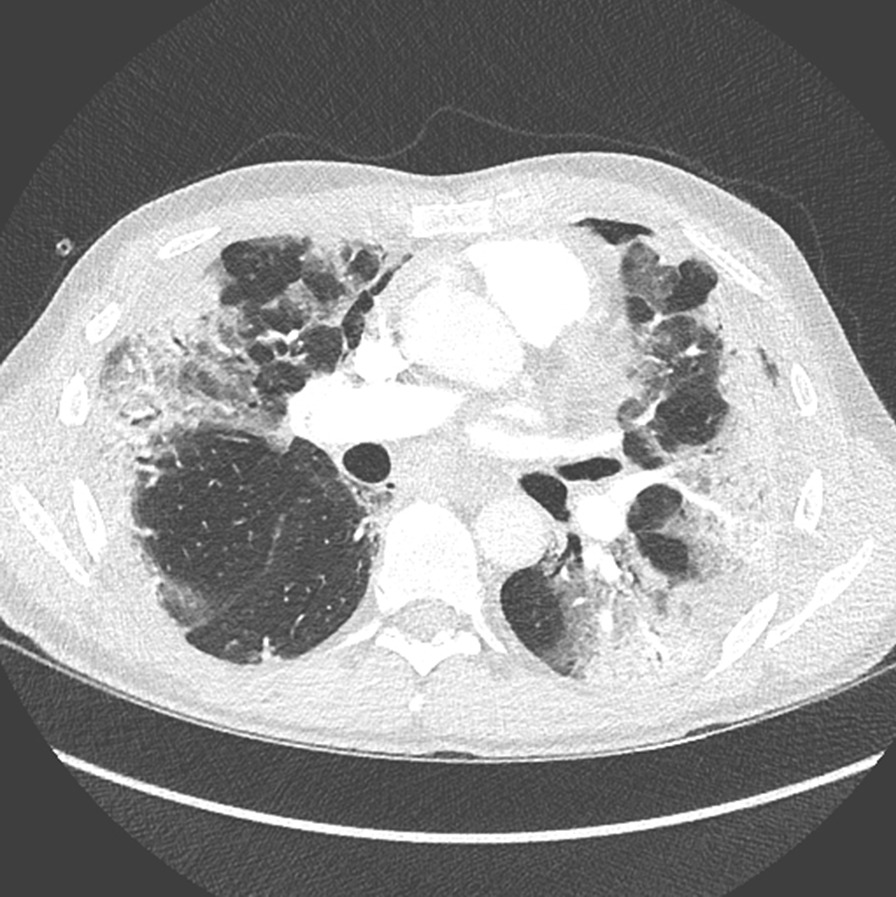
CT scan after 2 weeks of hospitalisation. In comparison with the initial CT scan, ground glass opacities have been replaced by much more extensive consolidation lesions, especially predominant at the bases. Majoration of bilateral consolidations present in the middle and inferior lobes

A second chest CT was performed 2 weeks after because of the lack of respiratory improvement, which showed replacement of the ground glass opacities with enlarged inferior consolidating aeras and a worsening course of disease (Fig. 2). As opportunistic infection was suspected, the patient underwent bronchoalveolar lavage and empirical antibiotics (Piperacillin/tazobactam 4 g, 4 times a day) was started.

Given the isolation of *Corynebacterium propinquum* on a quantitative culture 10^4^ colony forming unit /ml, antibiotics were continued for 7 days. Tocilizumab (8 mg/kg) and convalescent plasma (IgG titers > 1:320) were administered on day 10 and day 18 after admission respectively.

Nevertheless, the symptomatology of the patients didn’t improve and he still complained of breathlessness while sitting or standing and orthodeoxia was confirmed by SpO2 measurements (SpO2 98% on 5L/min O2 by Filtamask (FiO2 40%) in the supine position vs SpO2 89% on 5L/min O2 by Filtamask in the seated position).

**Fig. 3:**
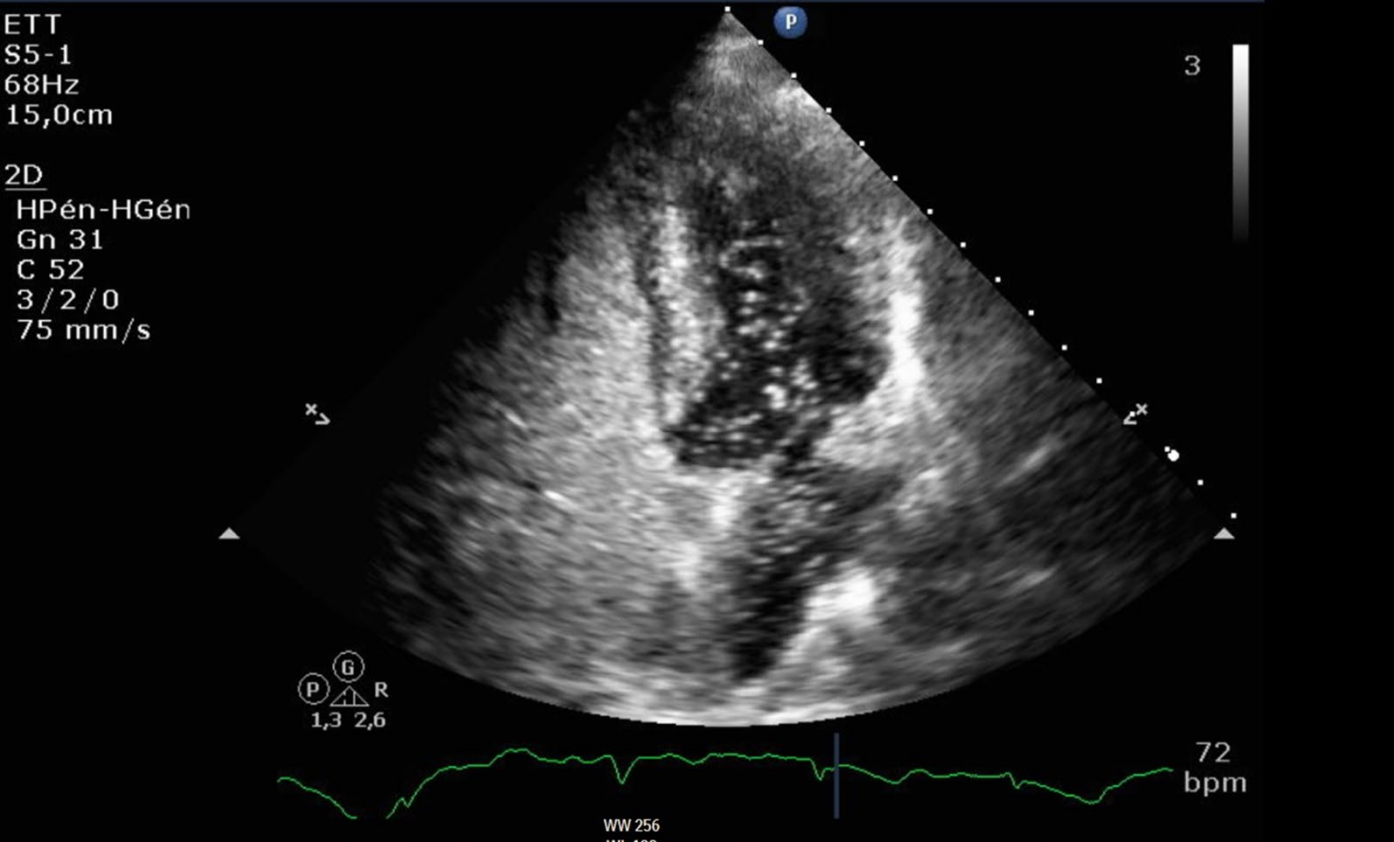
Bubble contrast echocardiography. Two-dimensional transthoracic echocardiography image showing a right-to-left shunt upon release of the Valsalva maneuver

Bubble-contrast transthoracic echocardiography revealed RTLS on Valsalva due to PFO (Fig. 3).

Progressively general condition favourably improved, the patient was discharged 1 week later with oxygen therapy (1 L/min). The arterial blood gas analysis showed PaO2 76 mmHg, PaCO2 38 mmHg, pH 7.46 and P/F ratio 316. Two weeks after discharge, orthodeoxia disappeared and SpO2 was 95% in supine position and 95% in standing position without oxygen therapy.

## Discussion

We report a case of POS which is a rare clinical syndrome defined by orthostatic dyspnoea and a quantified fall in arterial oxygen saturation of 5% or a PaO2 of 4 mmHg in upstand position [4, 6, 7]. Hypoxemia related to anatomical defect such as PFO requires a concomitant secondary functional dysfunction as pulmonary hypertension or increase of intrathoracic pressure (Valsalva) [8].

PFO is the most common congenital heart abnormality of foetal origin [5]. 25% of the adult population have a PFO which is not harmful in general as it was the case in our patient who performed a lot of sport activities [9]. Nonetheless, under pathologic conditions, PFO is capable to bypassing the pulmonary circulation as blood flow goes directly from the right to left atrium [5]. A number of pathological conditions have been associated with PFO such as stroke, or POS [9].

In ARDS, V/Q mismatch and right-to-left intrapulmonary shunting lead to hypoxaemia [10].

ARDS caused by SARS-CoV-2 is atypical and lead to different phenotypes in COVID-19 patients [1, 2]. Hypoxaemia in COVID-19 patients is not fully understood and seems to result from several pathogenic mechanisms such as alteration of hypoxic pulmonary vasoconstriction (HPV), coagulopathy and V/Q mismatch [11]. Lower lobes are commonly afflicted in COVID-19 patients and these are the gravitationally dependent lung compartments in upright positioning which is in favour of occurring of orthodeoxia also [11].

In this case, the patient presented several causes contributing to RTLS and POS.

First, in this case of unknown PFO, right-to-left interatrial shunt (RTLIAS) clearly can exacerbate hypoxaemia in a patient with COVID-19 pneumonia. In presence of abnormal elevated right atrial pressure caused by COVID-19 pneumonia, blood can pass across the interatrial communication [12].

Second, hypoxic lung diseases such as COVID-19 pneumonia is characterized by V/Q mismatch with regional differences in apical and basal regions of the lungs. In upright position, apical regions of the lungs act like a dead space, increasing V/Q mismatch and leading to a physiologic shunt and to POS. Furthermore, diffuse vascular damages and coagulopathy induced by SARS-CoV-2 disturb physiologic regulation of HPV and increases V/Q mismatch. Injured basal lungs are then pathologically hyperperfused in COVID-19 patients and contribute also to hypoxemia [11].

RTLIAS such as PFO is not the only cause of POS and other mechanisms that participate in decrease in lung oxygenation are also implicated in this case. Intrapulmonary shunt caused by SARS-CoV-2 pneumonia certainly contributed to refractory hypoxemia as well as V/Q mismatch. But the pulmonary hypertension due to HPV could also have triggered RTLS in a patient with an interatrial defect. Therefore, in case of COVID-19 patient with unexplained POS, the existence of PFO must be investigated.

## Data Availability

Not applicable.

## Abbreviations

ARDS: Acute respiratory distress syndrome
BAL: Bronchoalveolar lavage:
BCPAP: Boussignac continuous positive airway pressure
COVID-19: Coronavirus disease 2019
CPAP: Continuous positive airway pressure
CT: Computed tomography
ECG: Electrocardiogram
FiO2: Fraction of inspired oxygen
HPV: Hypoxic pulmonary vasoconstriction
PaO2: Partial pressure of oxygen
PaCO2: Partial pressure of carbon dioxide
PEEP: Positive End Expiratory Pressure
PFO: Patent foramen ovale
pH: Potential of hydrogen
POS: Platypnea-orthodeoxia syndrome
RTLIAS: Right-to-left interatrial shunt
RTLS: Right-to-left shunting
RT-PCR: Reverse transcriptase polymerase chain reaction
SARS-CoV-2: Severe acute respiratory syndrome coronavirus 2
SpO2: Peripheral oxygen saturation
V/Q: Ventilation–perfusion
